# Effects of Perinuclear Chromosome Tethers in the Telomeric *URA3*/5FOA System Reflect Changes to Gene Silencing and not Nucleotide Metabolism

**DOI:** 10.3389/fgene.2012.00144

**Published:** 2012-08-02

**Authors:** Betty P. K. Poon, Karim Mekhail

**Affiliations:** ^1^Faculty of Medicine, Department of Laboratory Medicine and Pathobiology, University of TorontoToronto, ON, Canada; ^2^Canada Research Chairs Program, Faculty of Medicine, University of TorontoToronto, ON, Canada

**Keywords:** SIR, cohibin, Esc1, Mps3, Heh1, telomere position effect, ribonucleotide reductase, URA3/5FOA

## Abstract

Telomeres are repetitive DNA sequences that protect the ends of linear chromosomes. Telomeres also recruit histone deacetylase complexes that can then spread along chromosome arms and repress the expression of subtelomeric genes in a process known as telomere position effect (TPE). In the budding yeast *Saccharomyces cerevisiae*, association of telomeres with the nuclear envelope is thought to promote TPE by increasing the local concentration of histone deacetylase complexes at chromosome ends. Importantly, our understanding of TPE stems primarily from studies that employed marker genes inserted within yeast subtelomeres. In particular, the prototrophic marker *URA3* is commonly used to assay TPE by negative selection on media supplemented with 5-fluoro-orotic acid (5FOA). Recent findings suggested that decreased growth on 5FOA-containing media may not always indicate increased expression of a telomeric *URA3* reporter, but can rather reflect an increase in ribonucleotide reductase (RNR) function and nucleotide metabolism. Thus, we set out to test if the 5FOA sensitivity of subtelomeric *URA3*-harboring cells in which we deleted various factors implicated in perinuclear telomere tethering reflects changes to TPE and/or RNR. We report that RNR inhibition restores 5FOA resistance to cells lacking RNR regulatory factors but not any of the major telomere tethering and silencing factors, including Sir2, cohibin, Mps3, Heh1, and Esc1. In addition, we find that the disruption of tethering pathways in which these factors participate increases the level of *URA3* transcripts originating from the telomeric reporter gene and abrogates silencing of subtelomeric *HIS3* reporter genes without altering RNR gene expression. Thus, increased 5FOA sensitivity of telomeric *URA3*-harboring cells deficient in telomere tethers reflects the dysregulation of TPE but not RNR. This is key to understanding relationships between telomere positioning, chromatin silencing, and lifespan.

## Introduction

Telomeres, which are repetitive DNA sequences at the ends of linear chromosomes, maintain genome stability and modulate gene expression. In the budding yeast *Saccharomyces cerevisiae*, telomeres mediate the recruitment of the Silent Information Regulator (SIR) complex, which is composed of the histone deacetylase Sir2 as well as the adapter proteins Sir3 and Sir4 (Longtine et al., 1989; Moretti et al., 1994; Buck and Shore, 1995; Imai et al., 2000; Moazed, 2001b). Sir2-dependent deacetylation of histone tails on nearby subtelomeric nucleosomes promotes the recruitment of additional SIR complexes. Iterative cycles of histone deacetylation and SIR recruitment promote the spreading of compact silent chromatin structures limiting access to RNA polymerase II and silencing genes within subtelomeric regions along chromosome arms (Gottschling et al., 1990; Moazed, 2001a). This reversible and heritable gene silencing process is known as telomere position effect (TPE) or telomeric silencing (Gottschling et al., 1990; Moazed, 2001a). The histone acetyltransferase Sas2 opposes the indefinite spreading of SIR complexes to more internal locations along the chromosome resulting in a gradient of telomeric silencing in which TPE is strongest right next to telomeres and gradually weakens as the distance to telomeres increases (Suka et al., 2002). Disruption of Sir2 or Sir3 significantly decreases replicative lifespan, which is the number of times a mother cell buds to generate a daughter cell before reaching senescence (Kaeberlein et al., 1999). Thus, telomeric maintenance and regulation of TPE by the SIR complex is crucial for the maintenance of replicative lifespan.

While it has only been recently recognized in mammals, TPE has been extensively studied in *S. cerevisiae* particularly through the use of reporter genes, such as *URA3*, inserted within subtelomeric chromosomal regions (Gottschling et al., 1990; Tennen et al., 2011). Counter selection of *URA3* expression on media supplemented with 5-fluoro-orotic acid (5FOA) constitutes a highly sensitive assay with a wide dynamic range for the assessment of changes to gene expression (Boeke et al., 1984; Gottschling et al., 1990). For example, wild-type cells, but not SIR-deficient cells, harboring a *URA3* reporter positioned proximal to the left arm telomere of chromosome VII (*URA3*-TELVII-L) can grow on 5FOA-containing media (Gottschling et al., 1990; Aparicio et al., 1991; Moazed, 2001a).

In *S. cerevisiae*, telomeres are clustered into 4–8 foci at the inner nuclear membrane (INM) and it is thought that perinuclear telomere anchoring and clustering maintains a high local concentration of SIR complexes to ensure efficient telomeric silencing (Maillet et al., 1996; Mekhail and Moazed, 2010; Chan et al., 2011). During the S phase of the cell cycle, telomere anchoring to the INM relies primarily on interactions between Sir4 and two major pathways, one implicating a protein called Esc1 (Establishes Silent Chromatin 1) and the other involving the SUN (Sad1-UNC-84) domain-containing protein Mps3 (MonoPolar Spindle 3; Andrulis et al., 2002; Bupp et al., 2007). Interestingly, Mps3 is itself implicated in at least two different perinuclear telomere anchoring processes, one implicating the enzyme telomerase and the other the Cohesin-related V-shaped cohibin complex, which is composed of Lrs4 and Csm1 (Antoniacci et al., 2007; Schober et al., 2009; Brito et al., 2010; Corbett et al., 2010; Wong, 2010; Chan et al., 2011). Specifically, cohibin is thought to link Sir4-bound telomeres to each other as well as to Mps3 and the LEM (Lap2β-Emerin-Man1) domain-containing INM protein Heh1 (Chan et al., 2011). While the deletion of SIR and cohibin proteins severely abrogates perinuclear telomere clustering and silencing, disruption of Esc1, and especially Mps3 or Heh1 leads to relatively mild phenotypes (Andrulis et al., 2002; Bupp et al., 2007; Grund et al., 2008; Schober et al., 2009; Corbett et al., 2010; Chan et al., 2011). Importantly, determining the relative impact of the various known telomere tethering/clustering factors in telomeric silencing assays, including those employing *URA3*-TELVII-L reporters, has been instrumental in identifying the above described contributions of these various factors within the perinuclear molecular networks regulating chromosome ends (Aparicio et al., 1991; Andrulis et al., 2002; Bupp et al., 2007; Grund et al., 2008; Chan et al., 2011).

However, recent findings suggest that increased 5FOA sensitivity in telomeric *URA3*-based assays may not always reflect disruptions to TPE but can rather reflect changes in nucleotide metabolism (Rossmann et al., 2011). In particular, deletions or mutations that alter the levels of ribonucleotide reductase (RNR), a complex that generates deoxyribonucleoside triphosphates needed for DNA synthesis, can increase 5FOA sensitivity even when TPE is unaffected (Rossmann et al., 2011).

Confidence in published data implicating the various known perinuclear telomere tethering factors in TPE remains high because most studies have assessed TPE via several approaches including the examination of endogenous subtelomeric gene expression as well as silent chromatin histone marks. However, it is unclear if the 5FOA sensitivities of cells lacking various telomere tethering/clustering factors in telomeric *URA3*/5FOA-based assays accurately reflect changes to TPE or rather possibly represent a combinatorial effect of changes to both TPE as well as nucleotide metabolism. Therefore, we set out to test these possibilities.

## Results

**Figure 1:**
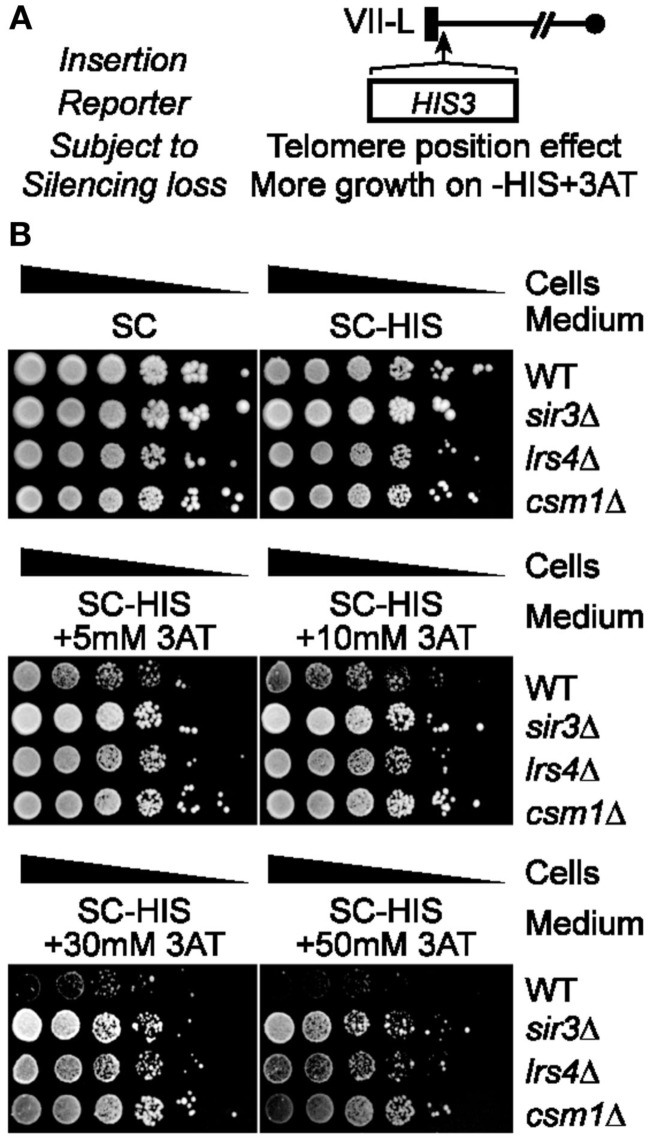
**Cohibin is required for silencing of the telomeric reporter gene *HIS3***. **(A)** Schematic of the *HIS3* reporter gene inserted proximal to TELVII-L. **(B)** Serial dilutions of cells with the telomeric *HIS3* reporter were plated on synthetic complete (SC) medium or SC media without histidine (−HIS) and with increasing concentrations of 3-amino-1,2,4-triazole (+3AT; *HIS3* silencing inhibits growth). WT, wild-type. Strains: KMY984, KMY986, KMY1303, KMY1307.

Silent Information Regulator and cohibin complexes are required for the establishment of endogenous silent chromatin marks and the silencing of endogenous subtelomeric genes (Aparicio et al., 1991; Chan et al., 2011). In addition, both SIR and cohibin proteins are also thought to be required for the silencing of the exogenous reporter genes *URA3* and *ADE2* inserted within subtelomeric regions (Gottschling et al., 1990; Chan et al., 2011). However, some mutations can hyper-activate RNR function and lead to a false loss-of-silencing in assays relying on the telomeric *URA3* reporter for the assessment of TPE on 5FOA (Rossmann et al., 2011). In addition, the expression of *URA3* and *ADE2* genes may be linked in some mutants via purine-pyrimidine cross-regulation (Rossmann et al., 2011). Therefore, we first sought to monitor TPE via the use of the *HIS3* reporter gene, which is another prototrophic marker whose expression can be assessed in sensitive genetic assays without relying on 5FOA (Figure 1A; Rossmann et al., 2011). Loss of *HIS3* silencing can be positively selected for on media containing 3-amino-1,2,4-triazole (3AT), which is a competitive inhibitor of the *HIS3* gene product (Brennan and Struhl, 1980). Wild-type and other cells were grown on either non-selective media, media lacking histidine, and media lacking histidine but supplemented with increasing amounts of 3AT. Importantly, *sir3*Δ, *lrs4*Δ, and *csm1*Δ cells grew much more efficiently than wild-type cells on 3AT-containing media (Figure 1B). In addition, the difference in growth phenotypes of *sir3*Δ, *lrs4*Δ, or *csm1*Δ relative to wild-type cells steadily increased in a 3AT dose-dependent fashion (Figure 1B). These results are consistent with previously published data revealing that cohibin proteins are required for the silencing of several endogenous subtelomeric genes as well as the SIR-dependent deacetylation of endogenous histones at chromosome ends (Chan et al., 2011). Thus, collectively, these findings demonstrate that SIR and cohibin are required for the silencing of the *HIS3*-TELVII-L reporter gene and suggest that results obtained via the use of *URA3* or other exogenous reporter genes may indeed reflect changes to TPE and not RNR function, although this remained to be directly tested.

**Figure 2:**
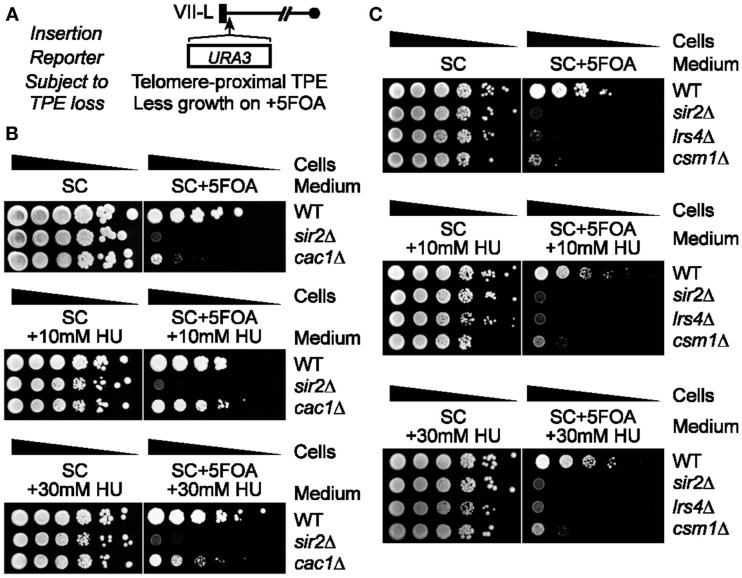
**Pharmacological inhibition of RNR function restores FOA-resistance in *cac1*Δ cells but not Sir2- or cohibin-deficient cells harboring a telomeric *URA3* reporter gene**. **(A)** Schematic of the *URA3* reporter gene inserted proximal to TELVII-L. **(B,C)** Serial dilutions of cells with the telomeric *URA3* reporter were plated on synthetic complete (SC) medium or medium supplemented with sublethal concentrations of hydroxyurea (+HU) and/or 5FOA (+5FOA). WT, wild-type. Strains: KMY368, KMY416, KMY1465, KMY74, KMY77.

Thus, we next set out to test if RNR inhibition affects the 5FOA sensitivity of *URA3*-TELVII-L cells lacking the major telomere silencing protein Sir2 (Figure 2A; Aparicio et al., 1991; Palladino et al., 1993). RNR inhibition was able to rescue the growth of Pol30 or Dot1 mutants, which were originally thought to be involved in TPE maintenance based on the telomeric *URA3* assay, but it was later discovered that these mutants did not have a general telomere silencing defect (Rossmann et al., 2011; Takahashi et al., 2011). In particular, pharmacological inhibition of RNR function via the addition of sublethal concentrations of hydroxyurea (HU) was able to restore 5FOA resistance to *pol30-8* cells (Rossmann et al., 2011). In addition, Pol30 physically interacts with Chromatin Assembly Factor-1 (CAF-1; consisting of Cac1, Cac2, and Cac3), which is a histone chaperone complex (Moggs et al., 2000). Disruption of CAF-1also increases RNR and hyper-sensitizes cells to 5FOA leading to a false loss-of-silencing in telomeric *URA3*/5FOA silencing assays (Rossmann et al., 2011). Consistent with these findings, FOA resistance can be restored to *cac1*Δ cells via the use of HU (Figure 2B; Rossmann et al., 2011). In contrast, we found that the FOA sensitivity of *sir2*Δ cells was unaltered by HU treatments (Figure 2B). Cohibin is thought to promote telomeric silencing at least in part by promoting perinuclear telomere clustering thereby increasing the local concentration of Sir2 at chromosome ends (Chan et al., 2011). This notion is based in part on ChIP data revealing that although SIR proteins were required to recruit cohibin to telomeres, loss of cohibin in turn reduced Sir2 concentrations at telomeres (Chan et al., 2011). These data point to a putative model for the generation of telomere clusters, where low amounts of SIR proteins bound at telomeres first recruit cohibin complexes (Poon and Mekhail, 2011). Cohibin complexes then start to cluster telomeres thereby increasing the local concentration of SIR proteins, which in turn recruits more cohibin complexes, and the cycle continues until perinuclear telomere clustering is complete (Poon and Mekhail, 2011). Consistent with the notion that cohibin acts through SIR to maintain telomere silencing, the 5FOA sensitivity of cells lacking cohibin subunits, Lrs4 or Csm1, similar to *sir2*Δ cells, was unaffected by HU treatment (Figure 2C). The growth rates of wild-type cells as well as cells lacking Cac1, Sir2, or cohibin proteins on HU-containing but 5FOA-free media were similar indicating that the concentrations of HU used are not indiscriminately affecting overall cellular growth, as expected (Figures 2B,C; Rossmann et al., 2011).

**Figure 3:**
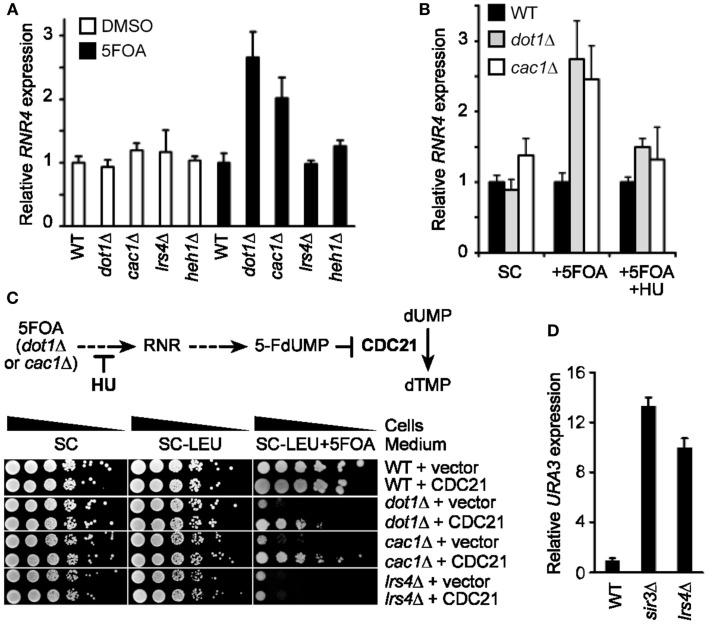
**FOA-treatment increases RNR expression in *dot1*Δ and *cac1*Δ cells, but not *lrs4*Δ cells, and *CDC21* overexpression or HU treatment restores 5FOA resistance to *dot1*Δ and *cac1*Δ cells, but not *lrs4*Δ cells**. **(A)** *RNR4* transcript levels (normalized to *ACT1*) as measured by quantitative reverse transcriptase PCR (qRT-PCR) following a 4-h treatment with 5FOA or DMSO. Error bars represent the SEM for three independent experiments. WT, wild-type. Strains: KMY368, KMY1391, KMY1465, KMY74, KMY59. **(B)** Expression levels of *RNR4* (normalized to *ACT1*) measured by qRT-PCR following a 4-h treatment with 5FOA with or without HU. Error bars represent the SEM for two independent experiments. WT, wild-type. Strains: KMY368, KMY1391, KMY1465. **(C)** Schematic showing impact of 5FOA on RNR in *dot1*Δ or *cac1*Δ cells (top). Serial dilutions of cells with the *URA3* reporter gene inserted proximal to telomere VII-L were plated on synthetic complete (SC) medium, medium lacking leucine (−LEU), or medium lacking leucine, and supplemented with 5FOA (−LEU + 5FOA; bottom). All cells were spotted on the same corresponding plates. WT, wild-type. Strains: KMY368, KMY1391, KMY1465, KMY74. **(D)** *URA3*-TELVII-L transcript levels (normalized to *ACT1*) as measured by qRT-PCR. Error bars represent the SEM for three independent runs. WT, wild-type. Strains: KMY1565, KMY1567, KMY1568.

Together, our results suggest that the 5FOA sensitivity of telomeric *URA3*-haboring cells that lack SIR or cohibin proteins reflects changes to TPE and is not due to hyperactivation of RNR. It was previously shown that the treatment of some mutants with 5FOA can increase RNR gene expression (Rossmann et al., 2011). In particular, Pol30 mutants exhibited about a threefold increase in *RNR4* transcript levels after a 4 h 5FOA treatment and it is thought that this increase, coupled to a mild increase in *URA3* expression in these mutants, induces 5FOA sensitivity in Pol30-deficient cells (Rossmann et al., 2011). We found that 5FOA treatment increases *RNR4* transcript levels in *dot1*Δ and *cac1*Δ cells, but not wild-type, *lrs4*Δ, or *heh1*Δ cells (Figure 3A). In addition, HU treatment abolished the 5FOA-induced increase in *RNR4* expression typically observed in *dot1*Δ and *cac1*Δ cells (Figure 3B). *CDC21* encodes the enzyme thymidylate synthase, which catalyzes the conversion of dUMP to dTMP within the RNR pathway (Figure 3C; Rossmann et al., 2011). In fact, 5FOA-induced changes to RNR gene expression repress Cdc21 and consequently dTMP generation causing a disruption of nucleotide metabolism. Consistent with this, we found that *CDC21* overexpression, similar to the RNR-inhibiting HU treatments discussed above, was able to rescue the growth of *dot1*Δ or *cac1*Δ cells, but not *lrs4*Δ cells, on 5FOA-containing media (Figure 3C; Rossmann et al., 2011). In addition, qRT-PCR analysis revealed that the expression of *URA3* at TELVII-L was indeed increased in *lrs4*Δ and *sir3*Δ cells (Figure 3D; Chan et al., 2011). Taken together, these results indicate that the 5FOA sensitivity of cells deficient in cohibin-dependent telomere tethering, but not *dot1*Δ or *cac1*Δ cells, does indeed reflect changes to TPE, but not nucleotide metabolism. In addition, these findings indicate that RNR-inhibiting HU treatments can be used to evaluate the TPE-dependent/RNR-independent contribution of various telomeric factors, such as cohibin complexes, to 5FOA resistance in telomeric *URA3* reporter gene assays.

**Figure 4:**
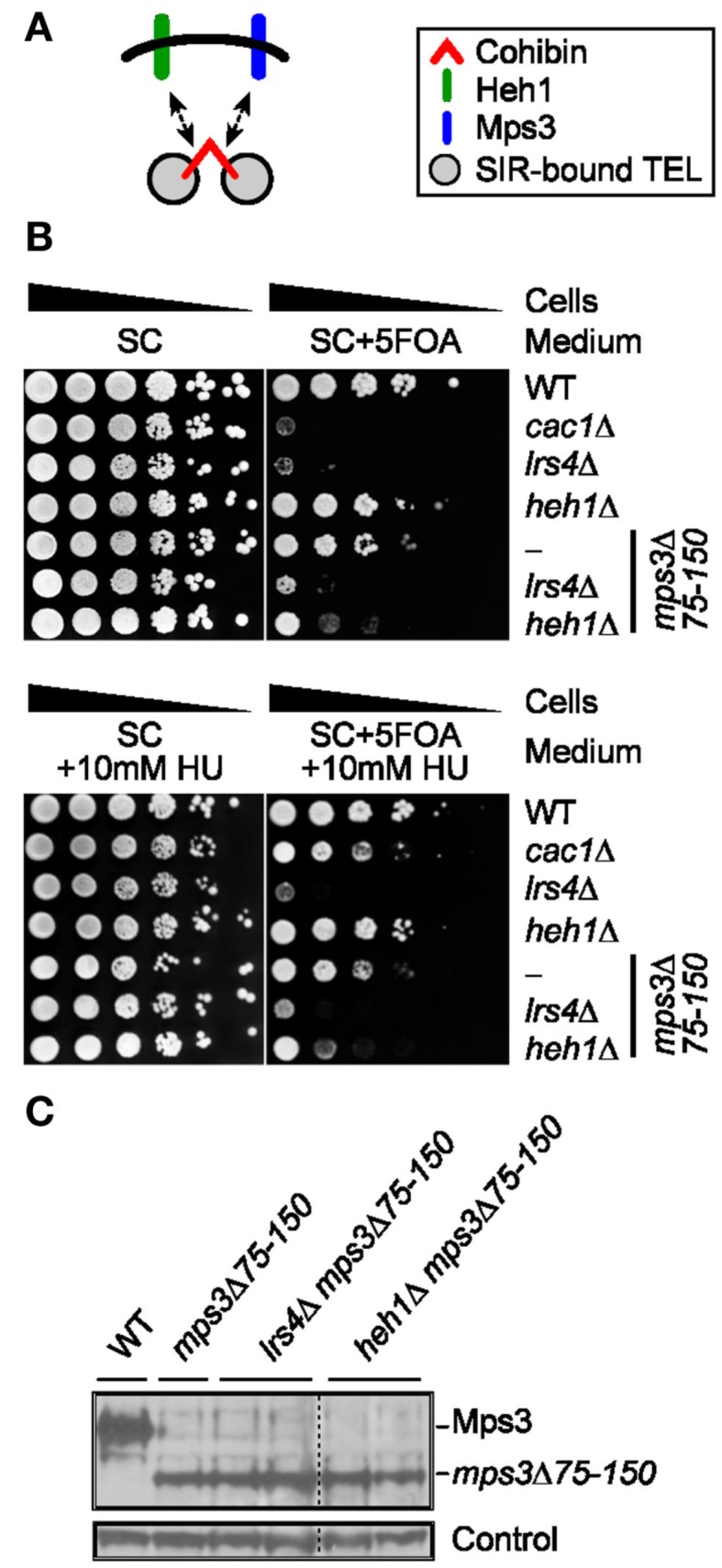
**Pharmacological inhibition of RNR function does not restore FOA-resistance in cells lacking cohibin-associated/telomere tethering nuclear envelope proteins**. **(A)** Schematic showing relationship between cohibin and the INM proteins Heh1 and Mps3 in perinuclear telomere tethering. **(B)** Serial dilutions of cells with the *URA3* reporter gene inserted proximal to telomere VII-L were plated on synthetic complete (SC) medium or medium supplemented with a sublethal concentration of hydroxyurea (+HU) and/or 5FOA (+5FOA). WT, wild-type. Strains: KMY368, KMY1465, KMY74, KMY59, KMY999, KMY1468, KMY1470. **(C)** Deletion of *LRS4* or *HEH1* does not affect *mps3*Δ75-150 levels as indicated by immunoblotting. A non-specific band served as loading control. All lanes were run and transferred from the same gel. WT, wild-type. Strains: KMY368, KMY999, KMY1467, KMY1468, KMY1469, KMY1470.

Cohibin complexes are thought to cooperate with both of the INM proteins Heh1 and Mps3 to mediate perinuclear telomere tethering and silencing (Figure 4A; Bupp et al., 2007; Grund et al., 2008; Chan et al., 2011). Consistent with this notion, 5FOA sensitivity in telomeric *URA3* assays is relatively weak for *mps3*Δ75-150 cells (Mps3 full length deletion is lethal) and negligible for *heh1*Δ cells, but the 5FOA sensitivity of *mps3*Δ75-150 *heh1*Δ cells is relatively higher and is closer to the sensitivity of *lrs4*Δ cells (Figure 4B). In addition, the sensitivity of *lrs4*Δ cells is similar to that of *mps3*Δ75-150 *lrs4*Δ cells indicating that Mps3 may operate at least in part through cohibin to ensure subtelomeric silencing (Figure 4B). Importantly, deletion of *LRS4* or *HEH1* did not change *mps3*Δ75-150 protein levels (Figure 4C). These results support a model in which Heh1 and Mps3 act through cohibin to ensure telomeric silencing (Chan et al., 2011). Thus, we asked if these 5FOA sensitivity phenotypes would be altered by RNR inhibition. Importantly, *cac1*Δ-rescuing/RNR-inhibiting sublethal concentrations of HU were unable to restore 5FOA resistance to *mps3*Δ75-150, *mps3*Δ75-150 *heh1*Δ, or *mps3*Δ75-150 *lrs4*Δ cells (Figure 4B). These results indicate that the differing sensitivities to 5FOA observed for these various genotypes indicate changes to TPE and not nucleotide metabolism.

**Figure 5:**
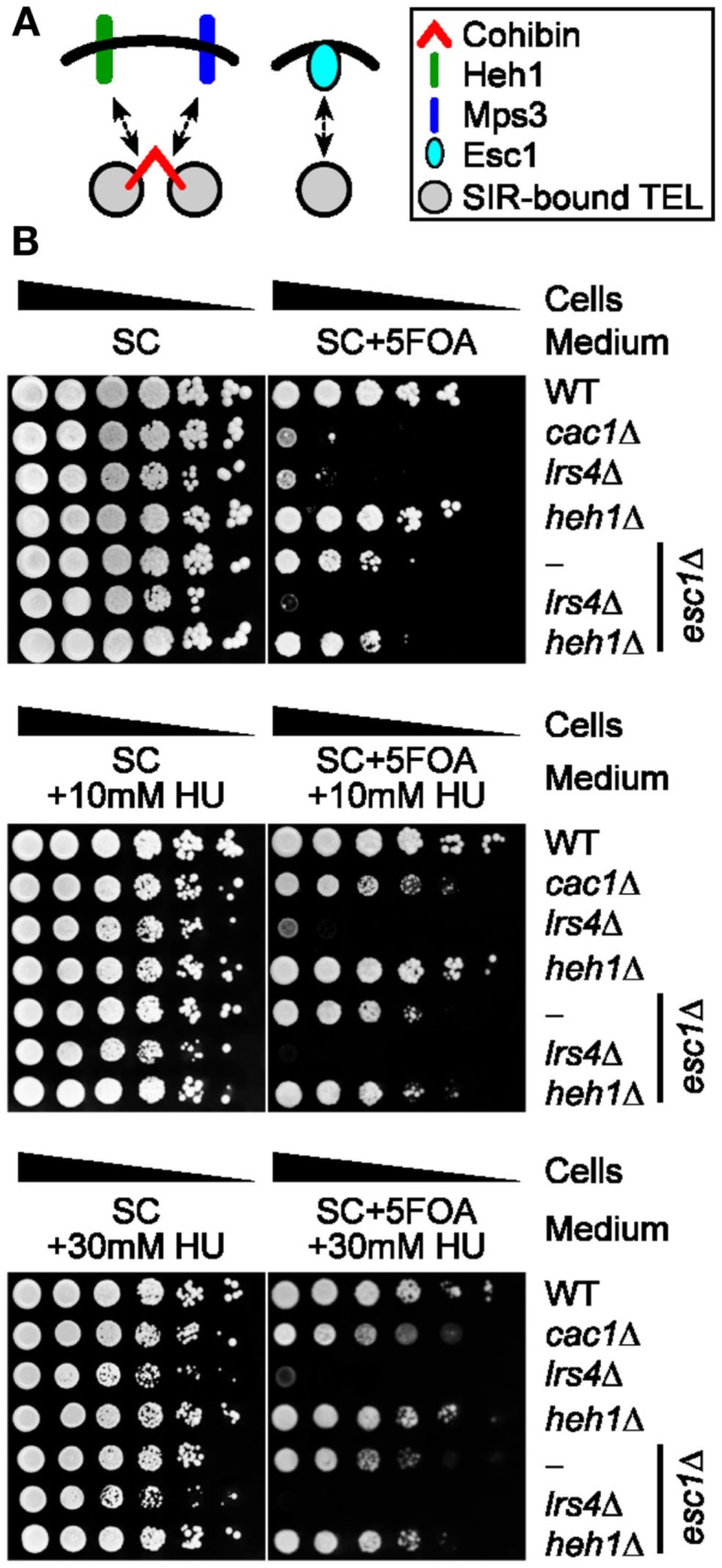
**Pharmacological inhibition of RNR function does not restore FOA-resistance in cells lacking Esc1 and/or cohibin-dependent telomere tethering**. **(A)** Schematic showing relationship between cohibin-dependent and Esc1-dependent perinuclear telomere tethering. **(B)** Serial dilutions of cells with the *URA3* reporter inserted proximal to telomere VII-L were plated on synthetic complete (SC) medium or medium supplemented with sublethal concentrations of hydroxyurea (+HU) and/or 5FOA (+5FOA). WT, wild-type. Strains: KMY368, KMY1465, KMY74, KMY59, KMY404, KMY492, KMY489.

Esc1 is another major factor that is thought to operate at least partly independently of cohibin to ensure perinuclear telomere tethering and silencing (Figure 5A; Andrulis et al., 2002; Chan et al., 2011). Consistent with this notion, although *lrs4*Δ cells are more sensitive to 5FOA than *esc1*Δ cells, the deletion of *ESC1* abolishes the residual low levels of 5FOA resistance typically observed in *lrs4*Δ cells indicating that cohibin and Esc1 can operate in parallel to promote telomeric silencing (Figure 5B; Chan et al., 2011). Thus, we tested if the 5FOA sensitivity profiles linked to these genotypes are affected by changes to RNR. Importantly, *cac1*Δ-rescuing/RNR-inhibiting sublethal concentrations of HU did not alter the 5FOA sensitivity profiles of *esc1*Δ, *esc1*Δ *heh1*Δ, or *esc1*Δ *lrs4*Δ cells (Figure 5B). These results confirm that Esc1 exerts a relatively small yet at least partly independent contribution relative to cohibin within the processes promoting telomeric silencing. Overall, our findings indicate that data obtained using the telomeric *URA3*/5FOA system indicate that Esc1, SIR, as well as cohibin complexes cooperating with the nuclear envelope proteins Mps3 and Heh1, are part of a perinuclear protein network that ensures TPE and controls gene expression patterns within subtelomeric regions independently of confounding RNR-related effects.

## Discussion

**Figure 6:**
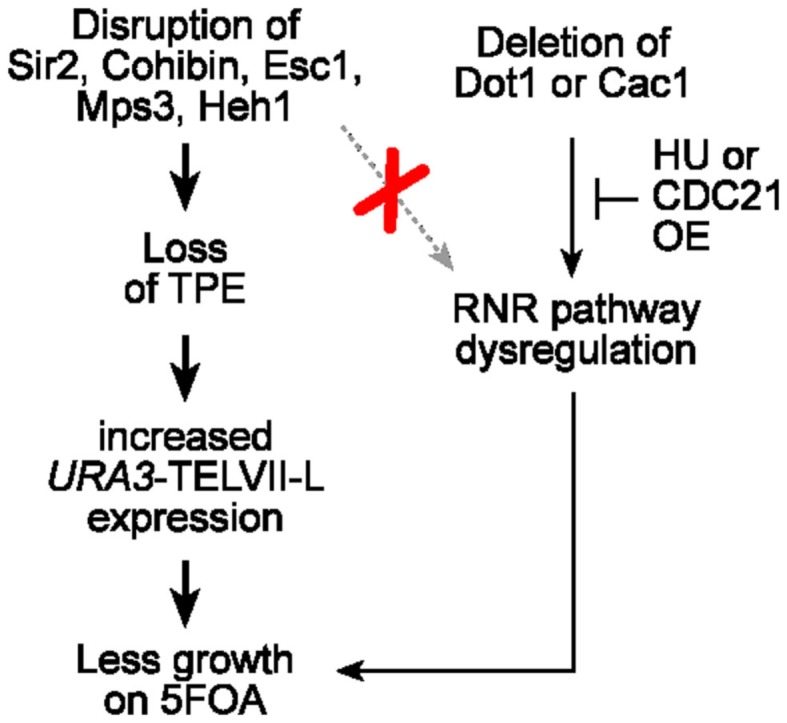
**Perinuclear telomere tethers impact the telomeric *URA3*/5FOA reporter system as a result of changes to TPE and not nucleotide metabolism**. Unlike *dot1*Δ or *cac1*Δ cells, RNR expression is unaffected in cells lacking various perinuclear telomere tethering factors including Sir2, cohibin, Esc1, Mps3, and Heh1. In addition, the 5FOA sensitivity of *URA3*-TELVII-L-harboring cells that lack these telomere-associated factors, but not Dot1/Cac1, is not restored upon RNR inhibition, which can be achieved via defined HU treatments or *CDC21* overexpression. Thus, the effects of major telomere tethering factors in telomeric *URA3*/5FOA reporter systems assaying for TPE reflect changes to chromatin assembly and gene expression but not nucleotide metabolism.

Our data reveal that the 5FOA sensitivity phenotypes observed for cells lacking one or combinations of telomere tethering/silencing factors, including SIR and cohibin complexes, Esc1, Mps3, and Heh1, are unaltered by RNR inhibition and that 5FOA treatment of these cells does not lead to increased RNR expression (Figure 6). Our results show that the observed phenotypes reflect changes to TPE and to the expression of the exogenous *URA3* reporter gene. In addition, we find that major telomere silencing factors such as SIR and cohibin proteins are required for the silencing of a subtelomeric *HIS3* reporter gene. Furthermore, our previous study showed that cells deficient in SIR or cohibin proteins had increased expression of an *ADE2* or *URA3* reporter gene inserted next to telomere V-R, indicating that the disruption of TPE is not specific to telomere VII-L (Chan et al., 2011). Moreover, similar results were obtained when the expression of endogenous subtelomeric genes located on various chromosomes was assessed (Chan et al., 2011). Thus, TPE is essentially abolished in cells lacking SIR proteins and is significantly weakened in cohibin-deficient cells. Together with previous studies, our findings indicate that individual INM proteins play a lesser role in ensuring TPE but additive effects are observed. Specifically, Mps3 and Heh1 seem to be operating at least partly through cohibin while Esc1 can operate at least partly independent of cohibin.

Our results are consistent with a model in which major telomere tethering and silencing factors, including SIR and cohibin complexes, Esc1, Mps3, and Heh1 play key roles within a perinuclear protein network ensuring telomere tethering and silencing (Figure 5A). The positioning of various factors within this network is based on results from protein–protein/DNA interactions, ChIPs examining silent chromatin marks, expression of endogenous subtelomeric genes, telomere localization studies, as well as the relative level of 5FOA sensitivity observed in telomeric *URA3*-harboring cells lacking one or more telomere-associated factors (Aparicio et al., 1991; Andrulis et al., 2002; Bupp et al., 2007; Grund et al., 2008; Chan et al., 2011).

Given that the pharmacological inhibition of RNR was unable to rescue the 5FOA sensitivity of cells lacking various telomere tethering and silencing factors, any possible effect that these factors may still have on nucleotide metabolism would be expected to be minor or insufficient to hyper-sensitize the cells enough to alter their growth on 5FOA. Indeed, we show that *RNR4* transcription is unchanged in cohibin-deficient cells upon treatment with 5FOA, consistent with the notion that the 5FOA sensitivity of cells lacking cohibin proteins is due to disruption of TPE. Thus, while we find that telomeric *URA3/*5FOA TPE assays incorporating pharmacological or genetic RNR inhibitors provide a very useful tool to sensitively dissect networks controlling TPE, we still are of the opinion that the latter should also be evaluated via the examination of endogenous subtelomeric reporter genes and histone marks as we previously reported (Chan et al., 2011). Genome-wide or multiple chromosome analyses of subtelomeric gene expression is important given that certain mutants may display a disruption of TPE that is telomere-specific (Takahashi et al., 2011). In particular, a previous study found that loss of telomeric silencing in *dot1*Δ cells was only observed at a handful of telomeres (Takahashi et al., 2011). Interestingly, the gene that showed the most severe loss-of-silencing in the absence of Dot1 was located on TELVII-L near the site of the *URA3* reporter gene that is commonly used to assess TPE in the *URA3*/5FOA silencing assay (Takahashi et al., 2011). This study demonstrates how mutants identified using the *URA3*/5FOA system may not display a general telomere silencing defect and exposes another weakness, in conjunction with RNR upregulation, in relying solely on the *URA3*/FOA system to identify proteins required for telomeric silencing.

Furthermore, overall cellular effects such as changes to replicative lifespan should also be examined in telomeric silencing mutants, given that the disruption of TPE and telomere maintenance leads to a decrease in replicative lifespan (Kaeberlein et al., 1999). Consistent with this notion, we previously showed that cohibin-deficient cells, which have lower concentrations of Sir2 at telomeres and display a strong loss of TPE across the genome, have decreased replicative life spans similar to that of Sir2 deficient cells (Chan et al., 2011). Increasing local telomeric Sir2 concentrations not only rescued telomeric silencing in *lrs4*Δ cells, but also rescued replicative lifespan defects linked to the disruption of TPE (Chan et al., 2011). This highlights the importance of regulating processes that affect telomeric SIR complex recruitment and consequently telomeric silencing in order to maintain replicative lifespan.

All in all, our work indicates that the effects of major telomere tethering and silencing factors on the 5FOA sensitivity of telomeric *URA3* reporter genes do reflect changes to TPE and not nucleotide metabolism. By providing key missing pieces of the puzzle of telomere regulation, this work highlights the important relationship between spatial genome organization, gene expression control, and cellular lifespan.

## Materials and Methods

### Strains and materials

Endogenous genes were deleted by PCR (Mekhail et al., 2008). The *mps3*Δ75-150 mutant was generated by cloning the *mps3*Δ75-150 transcript into PRS314 at the C-terminus of TRP using Pst-I and Sal-I restriction enzymes. Positive clones were confirmed by plasmid digestion and standard DNA sequencing. Yeast strains are listed in Table 1. Plasmids are listed in Table 2. Primers used in this study are listed in Table 3. Plasmids pKM133 and pKM135 were a kind gift from B. Stillman (Rossmann et al., 2011). The anti-Mps3/Nep98 antibody was a kind gift from S. Nishikawa and T. Endo (Nishikawa et al., 2003).

**Table 1:** **List of strains used in this study**.

Strain	Genotype	Source/reference
KMY368	W303a (ade2-1 can1-100 his3-11 leu2-3,112 trp1 ura3-1 GAL) TELVII-L::URA3 HMRΔE::TRP1	Gottschling et al. (1990), Buchberger et al. (2008)
KMY416	W303a (ade2-1 can1-100 his3-11 leu2-3,112 trp1 ura3-1 GAL) TELVII-L::URA3 HMRΔE::TRP1 sir2Δ::NATR	Buchberger et al. (2008)
KMY1465	W303a (ade2-1 can1-100 his3-11 leu2-3,112 trp1 ura3-1 GAL) TELVII-L::URA3 HMRΔE::TRP1cac1Δ::HPHR	This study
KMY74	W303a (ade2-1 can1-100 his3-11 leu2-3,112 trp1 ura3-1 GAL) TELVII-L::URA3 HMRΔE::TRP1 lrs4Δ::KANR	Chan et al. (2011)
KMY77	W303a (ade2-1 can1-100 his3-11 leu2-3,112 trp1 ura3-1 GAL) TELVII-L::URA3 HMRΔE::TRP1 csm1Δ::KANR	Chan et al. (2011)
KMY59	W303a (ade2-1 can1-100 his3-11 leu2-3,112 trp1 ura3-1 GAL) TELVII-L::URA3 HMRΔE::TRP1 heh1Δ::KANR	Chan et al. (2011)
KMY999	W303a (ade2-1 can1-100 his3-11 leu2-3,112 trp1 ura3-1 GAL) TELVII-L::URA3 HMRΔE::TRP1 mps3Δ::75-150-TRP	This study
KMY1467	W303a (ade2-1 can1-100 his3-11 leu2-3,112 trp1 ura3-1 GAL) TELVII-L::URA3 HMRΔE::TRP1 mps3Δ::75-150-TRP lrs4Δ::HPHR	This study
KMY1468	W303a (ade2-1 can1-100 his3-11 leu2-3,112 trp1 ura3-1 GAL) TELVII-L::URA3 HMRΔE::TRP1 mps3Δ::75-150-TRP lrs4Δ::HPHR	This study
KMY1469	W303a (ade2-1 can1-100 his3-11 leu2-3,112 trp1 ura3-1 GAL) TELVII-L::URA3 HMRΔE::TRP1 mps3Δ::75-150-TRP heh1Δ::HPHR	This study
KMY1470	W303a (ade2-1 can1-100 his3-11 leu2-3,112 trp1 ura3-1 GAL) TELVII-L::URA3 HMRΔE::TRP1 mps3Δ::75-150-TRP heh1Δ::HPHR	This study
KMY404	W303a (ade2-1 can1-100 his3-11 leu2-3,112 trp1 ura3-1 GAL) TELVII-L::URA3 HMRΔE::TRP1 esc1Δ::HPHR	Chan et al. (2011)
KMY492	W303a (ade2-1 can1-100 his3-11 leu2-3,112 trp1 ura3-1 GAL) TELVII-L::URA3 HMRΔE::TRP1 lrs4Δ::KANR esc1Δ::HPHR	Chan et al. (2011)
KMY489	W303a (ade2-1 can1-100 his3-11 leu2-3,112 trp1 ura3-1 GAL) TELVII-L::URA3 HMRΔE::TRP1 heh1Δ::KANR esc1Δ::HPHR	Chan et al. (2011)
KMY984	W303α his3Δ::NATMX4 adh4Δ::URA3-HIS3-VII-L (spore 20-3)	Rossmann et al. (2011)
KMY986	W303α sir3Δ::KANMX6 his3Δ::natMX4 adh4Δ::URA3-HIS3-VII-L (spore 4-1)	Rossmann et al. (2011)
KMY1303	W303α his3Δ::NATMX4 adh4Δ::URA3-HIS3-VII-L (spore 20-3) lrs4Δ::KANR	This study
KMY1307	W303α his3Δ::NATMX4 adh4Δ::URA3-HIS3-VII-L (spore 20-3) csm1Δ::KANR	This study
KMY1565	W303a (ade2-1 can1-100 his3-11 leu2-3,112 trp1 GAL) TELVII-L::URA3 HMRΔE::TRP1 ura3Δ::HPHR	This study
KMY1567	W303a (ade2-1 can1-100 his3-11 leu2-3,112 trp1 GAL) TELVII-L::URA3 HMRΔE::TRP1 sir3Δ::KANR ura3Δ::HPHR	This study
KMY1568	W303a (ade2-1 can1-100 his3-11 leu2-3,112 trp1 GAL) TELVII-L::URA3 HMRΔE::TRP1 lrs4Δ::KANR ura3Δ::HPHR	This study

**Table 2:** **List of plasmids used in this study**.

Strain	Genotype	Source
pKM133	2 μm, LEU2	Rossmann et al. (2011)
pKM135	*CDC21*/pRS425 = ORF + 124 bp 5′ + 21 bp 3′	Rossmann et al. (2011)

**Table 3:** **List of primers used for quantitative RT-PCR**.

Location	Size (bp)	Sequence 1	Sequence 2
*URA3*	93	TAAAGGCATTATCCGCCAAG	CCCGCAGAGTACTGCAATTT
*RNR4*	94	GCTACCGCTGGTAAGACCAC	CCTCTTGTCGAATCCAATAC
*ACT1*	153	GCCTTCTACGTTTCCATCCA	ATTTCCTTGGATGGGGTAGC

### Silencing assays

Telomere VII-L *URA3* reporter cells were spotted in 10-fold serial dilutions onto SC, SC + 10 mM HU, SC + 30 mM HU, SC + 5FOA, SC + 5FOA + 10 mM HU, and SC + 5FOA + 30 mM HU media. For strains harboring the *HIS3* reporter at telomere VII-L, 10-fold serial dilutions were spotted on SC, SC-HIS, SC-HIS + 5 mM 3AT, SC-HIS + 10 mM 3AT, SC-HIS + 30 mM 3AT, and SC + 50 mM 3AT media (Rossmann et al., 2011). Following spotting of serial dilutions, cells were incubated at 30°C for 2–3 days.

### Whole cell protein preparation

Whole cell lysates were prepared as previously described (Chan et al., 2011). Briefly, cells (OD_600_ ≈ 1.0) were subjected to bead beating with an equal volume of silica beads and lysis buffer [50 mM HEPES-KOH pH7.5, 150 mM NaCl, 10% glycerol, 0.5% NP-40, 1 mM EDTA, complete tablet protease inhibitor (Roche), and 1 mM PMSF] for 2 × 30 s with an intermittent 2 min incubation on ice. Lysates were clarified by two consecutive rounds of centrifugation at 16,000 rcf for 5 and 15 min. Samples were sheared through a 26G1/2 needle and boiled at 95°C for 5 min prior to SDS-PAGE.

### Liquid 5FOA and/or HU treatments

Treatment of cells were conducted as previously described (Rossmann et al., 2011). Cells were cultured overnight in SC medium containing 20 mg/l uracil and then diluted 1:50 and grown to log phase (OD_600_ ≈ 1.0). After which, 20 ml of culture was taken for RNA extraction, while the remainder of the culture was split and treated with 100X 5FOA solution to a final concentration of 1 g/l or the equivalent amount of DMSO. For HU rescue experiments, HU was added to the corresponding cell cultures to a final concentration of 10 mM.

### RNA extraction

Total RNA was prepared from logarithmically growing cells (OD_600_ ≈ 1.0) via hot phenol extraction. Cells were centrifuged and resuspended in 400 μl of AE buffer (50 mM NaOAc pH 5.3 and 10 mM EDTA in 0.1% DEPC). Forty microliters of 10% SDS and 440 μl of acidic phenol (pH 4.5) was added to each sample and incubated at 65°C for 5 min. The samples were rapidly chilled in a dry ice/EtOH bath until phenol crystals appeared. The samples were then centrifuged for 2 min at max speed at 4°C, and the upper phase was transferred to fresh tubes. One volume of phenol:chloroform (pH 4.5) was added to each sample, followed by centrifugation, and transferring of the upper phase to a fresh tube. Forty microliters of 3 M NaOAc (pH 5.3) and 2.5 volumes of cold 100% EtOH was added to each tube prior to centrifugation to precipitate the RNA. The resultant RNA pellet was washed with 2.5 volumes of cold 80% EtOH. The pellet was left to dry and then resuspended in 0.1% DEPC and quantified. Subsequently, 100 μg of the precipitated RNA was cleaned-up using RNeasy Mini Kit (Qiagen) with on-column DNase digestion. 1 μg of total RNA was treated with 1 U DNase I (Invitrogen) to further remove genomic DNA contaminations.

### Quantitative reverse transcriptase PCR

A 20 μl reverse reaction was carried out using 10 mM dNTPs, 50 μM random non-amers (Sigma), 500 ng total RNA, 5× First-Strand Buffer (Invitrogen), 100 mM DTT, 40 U/μl RNase OUT (Invitrogen), and 200 U/μl M-MLV reverse transcriptase (Invitrogen) at 23°C for 10 min, 37°C for 60 min, and 70°C for 15 min. A 10 μl qPCR reaction using 2× Power SYBR Green PCR Master Mix (Applied Biosystems), 1 μM each forward and reverse primer, and 1 μl of cDNA prepared from the RT reaction. The primers used in this study are listed in Table 3.

## Conflict of Interest Statement

The authors declare that the research was conducted in the absence of any commercial or financial relationships that could be construed as a potential conflict of interest.
